# People perceive parasocial relationships to be effective at fulfilling emotional needs

**DOI:** 10.1038/s41598-024-58069-9

**Published:** 2024-04-08

**Authors:** Shaaba Lotun, Veronica M. Lamarche, Ana Matran-Fernandez, Gillian M. Sandstrom

**Affiliations:** 1 Department of Psychology, University of Essex, Colchester, CO4 3SQ UK; 2 Brain-Computer Interfaces and Neural Engineering Lab, School of Computer Science and Electronic Engineering, University of Essex, Colchester, CO4 3SQ UK; 3 School of Psychology, University of Sussex, Brighton, Sussex, BN1 9RH UK

**Keywords:** Parasocial relationships, Emotion regulation, Responsiveness, Social media, Self-esteem, Psychology, Human behaviour

## Abstract

People regularly form one-sided, “parasocial” relationships (PSRs) with targets incapable of returning the sentiment. Past work has shown that people engage with PSRs to support complex psychological needs (e.g., feeling less lonely after watching a favorite movie). However, we do not know how people rate these relationships relative to traditional two-sided relationships in terms of their effectiveness in supporting psychological needs. The current research (*N*_*total*_ = 3085) examined how PSRs help people fulfil emotion regulation needs. In Studies 1 and 2, participants felt that both their YouTube creator and non-YouTube creator PSRs were more effective at fulfilling their emotional needs than in-person acquaintances, albeit less effective than close others. In Study 3, people with high self-esteem thought PSRs would be responsive to their needs when their sociometer was activated, just as they do with two-sided relationships.

## Introduction

People have an impressive ability to form one-sided “parasocial” relationships (PSRs) with targets that are incapable of returning the sentiment: people anthropomorphize non-human agents to cope with feeling disconnected from real people^1^, and form a sense of connection with beings who are impossible (e.g., fictional figures) or near impossible (e.g., celebrities) to meet in real-life^2^. Historically, this ability was seen as a necessary evil for people struggling to have their needs fulfilled by “real” relationships^3,4^. However, more recent perspectives suggest that these one-sided relationships might not be last resorts, but rather valuable social resources that fulfil many of the same needs as two-sided connections with close others and contribute to well-being^5–7^. However, little is known regarding the extent to which people see their PSRs as an effective means of supporting their psychological needs relative to traditional in-person relationships (i.e., close others and acquaintances). As an initial attempt to address this gap in the parasocial relationship literature, the current research examines this question with respect to emotion regulation needs.

### Advantages of parasocial relationships

People form parasocial relationships with real and fictional figures they never directly interact with, such as television characters and celebrities^2,8,9^. These one-sided relationships are psychologically powerful, influencing thoughts, feelings, and behaviors in ways that mirror two-sided relationships with friends and family^4,5,10^. For example, watching a favorite television show can reduce feelings of loneliness^11^, and having a connection with a TV-star-turned-political candidate can affect who people vote for in elections^12^.

Importantly, although early research viewed PSRs as merely temporary surrogates for real in-person interaction (e.g.,^13^), more recent research suggests that PSRs may have advantages compared to typical relationships because they offer a more consistent social resource; PSRs might not be able to directly respond to someone’s personal needs (e.g., they cannot give you a hug, or console you with just the right words), but they are also incapable of rejecting, betraying, or permanently leaving someone. And even though the death of a PSR—whether a celebrity or fictional figure—can lead to real grief^14–16^, people can always revisit/reignite their relationship with the PSR by re-engaging with existing materials (e.g., re-watching a favorite show). PSRs are therefore resilient in ways that two-sided relationships are not. Thus, far from using PSRs as a compensatory form of connectedness, people may deliberately include PSRs in their social portfolios explicitly because they can fulfill complex psychological needs (e.g., safety, emotion regulation).

### (Para)Social influences on emotional need fulfilment

Despite feeling like internal experiences, emotions are inherently social in nature. People’s emotional responses are directly influenced by the real and imagined presence of close others^17,18^, and humans rely on others to help regulate their emotions and fulfil their emotional needs^19–21^. For example, when people feel socially rejected, seeing a close other as being motivated and responsive to their needs provides a sense of security^22^.

When it comes to emotion regulation, the size and diversity of someone’s support network affects their ability to thrive. Cheung and colleagues^19^ found that people who had specialized social networks in which different relationships fulfilled different emotional needs, had greater well-being than people with generalized social networks in which each relationship fulfilled multiple needs. When people have emotional needs that cannot be filled by their existing networks, one option is to fill the gap by building new two-sided relationships. However, it takes time to develop new relationships. Instead, people could look to their broader social portfolio, such as their existing PSRs, whom they can readily engage with through the vast world of social media and entertainment at their fingertips. Since PSRs exist primarily in the imagination^13^, people are likely to find an existing PSR that they can mobilize to meet an unfulfilled emotional need.

### Current research

We explored the extent to which people believe that their PSRs (with YouTube content creators and non-YouTube creators, such as fictional characters) and traditional two-sided relationships can help them fulfil emotion regulation needs (Studies 1 and 2). Further, we tested whether people can fluidly engage with PSRs to regulate their emotions following an acute emotional stressor: social rejection (Study 3). Specifically, we tested the following pre-registered hypotheses:

1. Studies 1 and 2: We hypothesized that strong in-person (i.e., two-sided) ties would be the most effective at fulfilling emotion regulation needs, followed by strong parasocial relationships, then weak in-person ties, then weak parasocial relationships.
2. Study 3: We hypothesized that people who had an emotional need activated (feeling socially rejected) would see a parasocial relationship as more responsive to their needs, compared to those who did not have an emotional need activated.

In Study 3, we also tested the hypothesis that, as with two-sided relationships, self-esteem might moderate the extent to which people engage with PSRs following an acute interpersonal threat that activates emotional needs. Although we pre-registered a moderation effect, we did not pre-register the directionality of the corresponding simple effects. We therefore treat the moderation as an exploratory hypothesis.

The datasets, pre-registrations, materials, analysis code, and Supplemental Online Material (SOM) are available on OSF: https://osf.io/epmvf. All methods were carried out in accordance with relevant guidelines and regulations. All experimental protocols received ethical approval from the University of Essex Ethics Committee. Informed consent was obtained from all participants at the start of each study.

## Study 1

Study 1 asked participants to consider the effectiveness of both strong and weak PSRs, in addition to strong and weak two-sided relationships. We expected to find that strong in-person ties were the most effective at fulfilling emotion regulation needs, followed by strong parasocial relationships, then weak in-person ties, then weak parasocial relationships (H1).

### Methods

#### Participants

Participants for Studies 1 and 3 were simultaneously reached through a group of ten influential creators who were paid up to £200 to promote the survey link in their YouTube videos. Participants in Studies 1 and 3 could enter a single draw to win one of four £100 gift vouchers. Creators emphasized that participation was completely voluntary and informed participants of the optional gift voucher draw. Participants aged 16–17 years were recruited into Study 1, which had ethical approval to recruit younger participants. Participants 18 years and older were randomly assigned to Study 1 or Study 3. The survey was closed one week after the surveys were promoted, after confirming that the sample size was adequate. There were 1,688 participants in Study 1 (1161 identified as female, 399 as male, 128 in another way), aged 16–78 years (*M*_age_ = 22 years, *SD* = 8). A sensitivity analysis (1 − β = 0.80, α = 0.05) suggests that our sample can detect effects of *f* ≥ 0.03.

#### Procedure

Participants nominated 2 two-sided relationships, adapting instructions from Sandstrom and Dunn^24^: a strong tie (“someone you are very close to, someone who you know really well (and knows you really well), and someone who you confide in or talk to about yourself, or your problems”) and a weak tie (“someone you are not very close to, who you don’t know very well (though you might consider them a friend), and would be unlikely to confide in”). They also nominated two YouTube creators: a strong PSR (“you watch and feel like you ‘know’ the most, and may want to confide in”), and a weak PSR (“you don’t know very well, and would be unlikely to want to confide in”). For each target, participants first completed the measure of emotional need fulfilment, then closeness and perceived responsiveness. Targets were presented in random order.

#### Measures

##### Emotional need fulfilment

Participants completed seven items from Cheung et al.^19^ (e.g., cheering up when sad, amplifying anger) for each target (two-sided strong tie, two-sided weak tie, strong PSR, weak PSR), using a 7-point scale (1 = *least effective*, 7 = *most effective;* αs = 0.81–0.90).

##### Relationship responsiveness and closeness

Believing that someone can help fulfil important self-regulatory needs should have implications for other interpersonal outcomes. Notably, people tend to feel closer to others who help them regulate and fulfil their emotional needs, and they anticipate that these close others will be responsive to their needs again in the future^24–26^. Therefore, we used a 12-item measure (adapted from^27^) to assess perceived responsiveness of each target (e.g., ‘[X] sees the ‘real’ me’; αs = 0.95–0.96). A 9-item measure (adapted from^28^) was used to assess closeness with each target (e.g., ‘I disclose important personal things to [X]’; αs = 0.91–0.92). Three items were removed from the original 12-item closeness measure because they did not fit the context of PSRs (see study materials on OSF). Both were assessed on a 7-point scale (1 = *strongly disagree,* 7 = *strongly agree*).

Additional measures were assessed (e.g., YouTube watching behaviors, need to belong, personality, social network size); see OSF for full materials. Of particular interest, we assessed well-being and self-esteem, and pre-registered analyses of these variables. However, since these analyses are not directly related to the primary research question examined in this paper, we have chosen to report results on these measures in the SOM.

### Results

**Figure 1:**
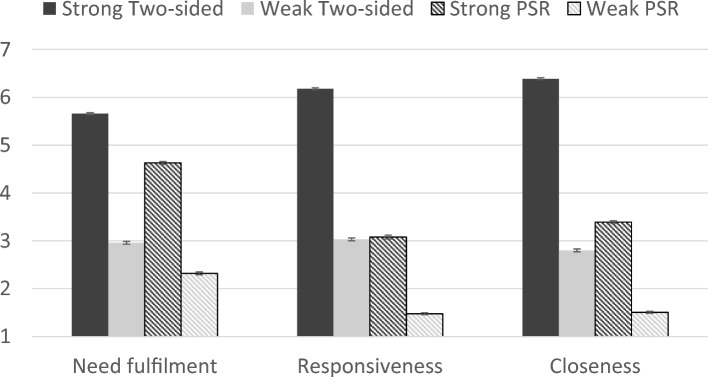
Differences between targets on perceived need fulfilment, responsiveness, and closeness for Study 1. Error bars reflect the standard error.

We investigated the extent to which people rated their strong and weak two-sided relationships and PSRs as effective for fulfilling their emotional needs. There were significant differences between targets in terms of perceived emotional need fulfilment, *F*(3, 4956) = 2,893.05, *p* < 0.001, ${\eta }_{p}^{2}$ = 0.64, perceived responsiveness, *F*(3, 4911) = 4,609.18, *p* < 0.001, ${\eta }_{p}^{2}$ = 0.74, and perceived closeness, *F*(3, 4935) = 6,342.06, *p* < 0.001, ${\eta }_{p}^{2}$ = 0.79 (see Fig. 1).

Consistent with our pre-registered hypothesis, strong PSRs were rated as significantly more effective than weak two-sided relationships at fulfilling emotional needs, *p* < 0.001, and were perceived as significantly closer, *p* < 0.001. However, contrary to our hypothesis, strong PSRs did not differ from weak two-sided relationships in perceived responsiveness, *p* = 0.32. Weak PSRs were rated as less effective at emotion regulation, and were perceived as less responsive and less close than all other targets, *p*s < 0.001. As hypothesized, strong two-sided relationships were rated as the most effective at fulfilling emotional needs and were perceived as more responsive and closer than all other targets, *p*s < 0.001.

We ran an earlier version of Study 1 that asked people to nominate strong, but not weak, PSRs. For conciseness, we report this pilot study as Study 0 in the SOM. The pattern of results broadly replicates the findings of Study 1, and a mini meta-analysis that includes this study in addition to Studies 1 and 2 further replicates the pattern (see SOM).

## Study 2

Study 2 aimed to replicate and establish the generalizability of Study 1 by 1) recruiting a sample that was more representative than its convenience sample of YouTube viewers, and 2) examining a broader range of types of PSRs (e.g., celebrities, fictional characters). As this was a direct replication, we had the same hypothesis as Study 1.

### Methods

#### Participants

We used Prolific Academic to recruit people from the U.S. and the U.K. who reported being fluent in English. Since the goal of this study was to establish generalizability, we aimed to recruit as many people as possible, subject to budgetary constraints, resulting in a target of 500 people. A total of 1087 participants completed pre-screening questions. Of these, 567 were eligible to complete the full survey, but due to a programming error, only 534 were offered this opportunity. The 501 who did so constitute our final sample (343 identified as female, 155 as male, 3 in another way; aged 18–70, *M*_age_ = 36, *SD* = 11). A sensitivity analysis (1 − β = 0.80, α = 0.05) suggests that our sample can detect effects of *f* ≥ 0.06.

#### Procedure

All participants responded to two-prescreening questions—assessing whether or not they had (1) a strong YouTube PSR and (2) a strong PSR of another kind (e.g., television, movie or book character, or celebrity). Only participants who reported having at least one kind of strong PSR were invited to complete the full survey.

For the full survey, participants nominated a strong and a weak two-sided relationship target. If they reported having a strong YouTube PSR, they were asked to nominate two YouTube creators: one strong and one weak. If they reported having a strong PSR, but not with a YouTube creator, they were asked to nominate a strong and a weak non-YouTube PSR (see materials on OSF for complete instructions). For each target, participants first completed the measure of emotional need fulfilment, then closeness and perceived responsiveness. Targets were presented in random order.

#### Measures

##### Emotional need fulfilment

We used the same measure of emotional need fulfilment as in Study 1, for each of the four targets (αs = 0.84–0.92).

##### Relationship responsiveness and closeness

We used the same measures of responsiveness (αs = 0.95–0.98) and closeness (αs = 0.92–0.94) as in Study 1.

We also assessed well-being and self-esteem; see the SOM for analyses of these variables.

### Results

The majority of participants (567 out of 1087; 52%) reported in the pre-screening questions that they had at least one type of strong PSR. A total of 387 participants (36%) had a strong PSR with a YouTube creator (age range 18–70, *M*_age_ = 35, *SD* = 11), whereas 437 participants (40%) had a strong non-YouTube PSR (age range 18–70, *M*_age_ = 37, *SD* = 11), and 257 participants (24%) had both kinds of strong PSRs.

Of the final sample of 501 who passed the pre-screening questions and completed the survey, 346 responded with respect to a YouTube PSR (235 of these also had a strong non-YouTube PSR), and 155 responded with respect to a non-YouTube PSR.

**Figure 2:**
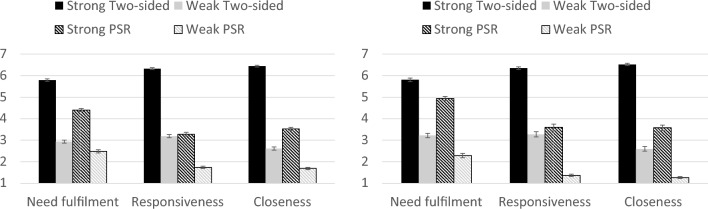
Differences between targets on perceived need fulfilment, responsiveness, and closeness for Study 2, for YouTube PSRs (left) and non-YouTube PSRs (right). Error bars reflect the standard error.

For both YouTube and non-YouTube PSRs, there were significant differences between targets in terms of perceived emotional need fulfilment, perceived responsiveness, and perceived closeness (see Table 1, Fig. 2). The results were identical to those in Study 1. Strong PSRs were rated as significantly more effective than weak two-sided relationships at fulfilling emotional needs and were perceived as significantly closer, *p’s* < 0.001, but did not differ significantly from them in terms of perceived responsiveness, *p’*s = 0.43 and 0.08. Weak PSRs were rated as less effective at emotion regulation, and were perceived as less responsive and less close than all other targets, *p’*s < 0.001. Strong two-sided relationships were rated as the most effective at fulfilling emotional needs and were perceived as more responsive and closer than all other targets, *p’*s < 0.001.

**Table 1:** ANOVA results for YouTube and non-YouTube PSRs for Study 2.

	YouTube PSR	Non-YouTube PSR
Perceived emotional need fulfilment	*F*(3, 1035) = 606.69, *p* < 0.001, ${\eta }_{p}^{2}$ = 0.64	*F*(3, 462) = 334.32, *p* < 0.001, ${\eta }_{p}^{2}$ = 0.69
Perceived responsiveness	*F*(3, 1035) = 955.92, *p* < 0.001, ${\eta }_{p}^{2}$ = 0.74	*F*(3, 462) = 409.74, *p* < 0.001, ${\eta }_{p}^{2}$ = 0.73
Perceived closeness	*F*(3, 1035) = 1,372.77, *p* < 0.001, ${\eta }_{p}^{2}$ = 0.80	*F*(3, 462) = 670.74, *p* < 0.001, ${\eta }_{p}^{2}$ = 0.81

## Study 3

In Studies 1–2, people believed their PSRs *could be* effective in regulating their emotions, and indeed believed they could be more responsive than weak two-sided relationships (i.e., acquaintances). This builds on previous work which suggests that people do engage with PSRs when they are experiencing certain emotions (e.g., loneliness;^11^). However, this previous work does not account for the psychological mechanisms behind why people engage with PSRs this way. Thus, the final aim of our studies was to integrate these two lines of research: assessing whether people are motivated to engage with PSRs when they have an emotional need acutely activated, expressly because they perceive their PSRs are able to fulfil these emotional needs.

In Study 3, we therefore extended our prior findings by activating people’s emotional regulatory needs through social rejection. The activation of the sociometer (i.e., a gauge of social acceptance;^29^) motivates people to seek interpersonal connection and approval. One way people accomplish this with their traditional (i.e., two-sided) relationships is by affirming that their relationships are responsive, and thus capable of meeting the needs signaled by the sociometer^30^. Thus, if people actively use PSRs to regulate their emotions in the same way, social threats should motivate people to report that their PSRs are responsive to the needs signaled by the sociometer. We hypothesized that people who had an emotional need activated (feeling socially rejected) would see a parasocial relationship as more responsive to their needs compared to those who did not have an emotional need activated (H2).

Additionally, there are important individual differences in how people engage with their social networks in response to their sociometers^30^. It is therefore equally possible that there are individual differences that moderate the extent to which people will turn to their PSRs to fulfil emotional needs. Thus, in Study 3, we tested self-esteem as a pre-registered moderator for the effects found in Studies 1–2. We anticipated a priori that people with high and low self-esteem may engage with PSRs differently following an interpersonal threat, because we know from past research that people’s acute responses to interpersonal threats differ depending on their self-esteem^30–33^. However, the direction of these effects was difficult to predict. People with low self-esteem typically experience interpersonal threats more acutely, and are more likely to distance from, or push away, their close others in times of need (i.e., perceive them as less responsive in their moment of need). However, we also know from past research that people with low self-esteem benefit from their PSRs to a greater extent than those with high self-esteem (e.g.,^34^). It may therefore be difficult to detect a difference in how people with low self-esteem perceive the responsiveness of their PSRs after a threat compared to when there is no threat, because they chronically perceive their PSRs as responsive. Thus, although we had a priori expectations that we would observe important differences in how people with high and low self-esteem rely on their PSRs in times of need, we did not pre-register hypotheses as to the directionality of these findings.

### Methods

#### Participants

From the sample strategy described in Study 1, 960 participants of at least 18 years of age were randomly assigned to Study 3 and completed all the dependent variables. After removing 64 responses due to non-compliance with instructions (see below for details), our final sample was 896 participants (663 identified as female, 172 as male, 61 in another way; aged 18–69, *M*_age_ = 24, *SD* = 8). A sensitivity analysis (1 − β = 0.80, α = 0.05) suggests that our sample can detect effects of f^2^ ≥ 0.01.

#### Procedure

Participants reported their self-esteem, and were then randomly assigned to write either about an experience where they felt hurt and disappointed by someone with whom they have a strong relationship (social threat condition; *N* = 435) or about an experience where they felt encouraged and supported by them (no threat condition; *N* = 461). This manipulation has been used to reliably elicit social rejection in the past (e.g.,^31,32^). Next, participants were asked to nominate the YouTube creator that they felt they knew the most (i.e., a strong PSR), and report how responsive they felt this target was to their needs, and how close they felt towards this target.

One of the authors reviewed participants’ descriptions of their recalled experience to determine compliance with the instructions for the threat manipulation. We removed from analyses participants who did not write anything (*n* = 61), or provided gibberish or nondescript responses (*n* = 3).

#### Measures

##### Self-esteem

Participants completed a 10-item measure of self-esteem (α = 0.91; Rosenberg, 1965), assessed on a 7-point scale (1 = *strongly disagree*, 7 = *strongly agree*).

##### Relationship responsiveness and closeness

The same measures from Studies 1–2 were used to assess perceived responsiveness of (α = 0.95) and closeness to (α = 0.88) the PSR.

Additional measures were assessed (e.g., YouTube watching behaviors, need to belong, personality, social network size); see OSF for full materials.

### Results

#### Perceived responsiveness

**Figure 3:**
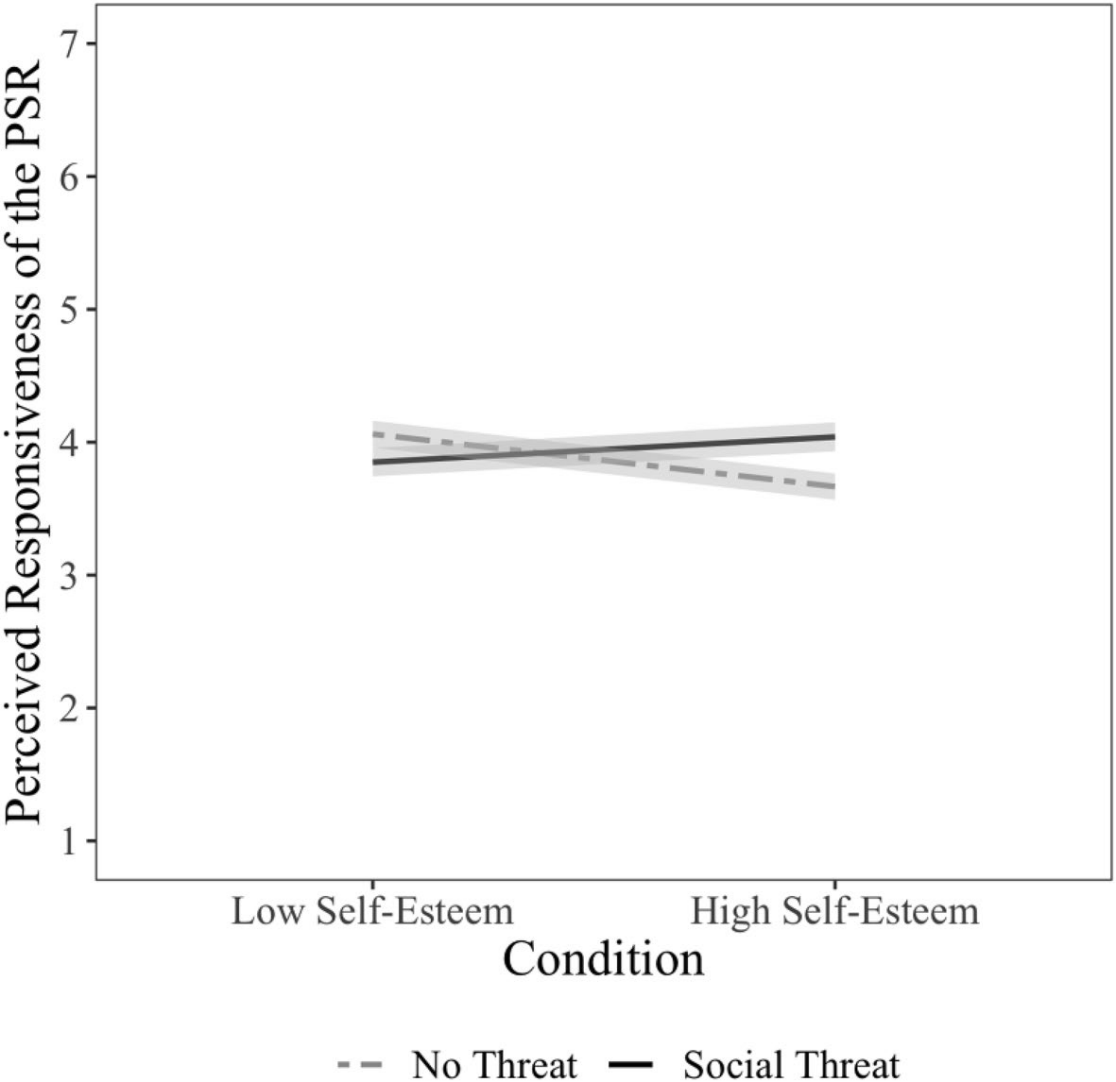
The condition by self-esteem interaction predicting perceived responsiveness of the PSR in Study 3. Self-esteem is mean centered. High and low self-esteem are plotted at ± 1 SD from the mean respectively.

We ran regression analyses predicting perceived responsiveness from (1) the main effects of social threat condition (1 = social threat, − 1 = no threat) and self-esteem (centered), and (2) their two-way interaction. Contrary to our hypothesis, the main effect of social threat condition was not significantly associated with perceived responsiveness, *b* = 0.08, CI_95_ = [− 0.12, 0.29], β = 0.03, *t*(893) = 0.79, *p* = 0.43, ${\eta }_{p}^{2}$ < 0.001, nor was the main effect of self-esteem, *b* = − 0.05, CI_95_ = [− 0.13, 0.03], β = − 0.04, *t*(893) =  − 1.31, *p* = 0.19, ${\eta }_{p}^{2}$ = 0.002, when the interaction term was not included in the model. However, the condition by self-esteem interaction was significant, *b* = 0.23, CI_95_ = [0.07, 0.39], β = 0.13, *t*(892) = 2.81, *p* = 0.01, ${\eta }_{p}^{2}$ = *0.*01 (see Fig. 3). We decomposed this interaction in two ways, to test for the simple effects of threat and of self-esteem.

##### The simple effects of social threat

People with high self-esteem (HSE; + 1*SD*) reported greater perceived responsiveness from their PSR when threatened relative to those who were not threatened, *b* = 0.37, CI_95_ = [0.09, 0.66], β = 0.12, *t*(892) = 2.55, *p* = 0.01, ${\eta }_{p}^{2}$ = 0.01. This is consistent with past work on close two-sided relationships, which shows that people with high self-esteem defensively seek evidence that their partners can be responsive to their needs when they are socially threatened (e.g.,^31,32^). However, unlike in past research on close two-sided relationships, people with low self-esteem (LSE; − 1 *SD*) did not engage in self-protection by perceiving less responsiveness from PSRs in the threat condition, relative to those who were not threatened, *b* = − 0.21, CI_95_ = [− 0.50, 0.08], β = − 0.07, *t*(892) =  − 1.44, *p* = 0.15, ${\eta }_{p}^{2}$ = 0.002.

##### The simple effects of self-esteem

The simple effect of self-esteem was not significant for people in the social threat condition, *b* = 0.08, CI_95_ = [− 0.05, 0.20], β = 0.06, *t*(892) = 1.23, *p* = 0.22, ${\eta }_{p}^{2}$ = 0.002, but it was significant in the no threat condition, *b* = − 0.16, CI_95_ = [− 0.26, − 0.05], β = − 0.13, *t*(892) =  − 2.85, *p* = 0.004, ${\eta }_{p}^{2}$ = *0.*01, such that people with *low* self-esteem (vs. high self-esteem) perceived their PSRs are more responsive to their needs.

In other words, when people feel threatened in their in-person social world, both LSEs and HSEs believe their PSRs are equally responsive. On the other hand, when their in-person social world appears to be capable of responding to their needs, people with low self-esteem perceive their PSRs as more responsive to their needs than people with higher self-esteem, consistent with past research finding that LSEs more consistently engage with PSRs (e.g.,^11,34^).

Although the moderated effects of self-esteem were pre-registered, it is important to note that we had not pre-registered the direction of the simple slopes. Some readers may therefore interpret these findings as exploratory, and therefore future research may want to replicate these findings with pre-registered directional hypotheses regarding the simple slopes.

#### Closeness

The same analytic strategy was used to test for closeness with the nominated PSR. Neither the main effect of condition, *b* = 0.04, CI_95_ = [− 0.13, 0.21], β = 0.02, *t*(893) = 0.46, *p* = 0.65, ${\eta }_{p}^{2}$ < 0.001, the main effect of self-esteem, *b* = − 0.06, CI_95_ = [− 0.13, 0.01], β = − 0.06, *t*(893) =  − 1.77, *p* = 0.08, ${\eta }_{p}^{2}$ = 0.004, or the condition by self-esteem interaction was significant, *b* = 0.09, CI_95_ = [− 0.04, 0.23], β = 0.06, *t*(892) = 1.36, *p* = 0.17, ${\eta }_{p}^{2}$ = *0.*002. In past work with two-sided relationships, HSEs typically defensively affirm closeness with a close other, as well as perceived responsiveness, in response to a relationship threat (e.g.,^35^). The fact that this pattern is not mirrored for PSRs suggests that there may be limitations on the emotional needs PSRs can realistically fulfill (e.g., the need to feel safe, but not the need for closeness/intimacy).

## General discussion

The present studies examined how effective people think their PSRs are, relative to their two-sided relationships, at fulfilling their emotional needs. Across three studies we found that people believe their “one-sided” PSRs can fulfil their emotional needs in ways that mirror their traditional two-sided social relationships with strong and weak ties. Although strong two-sided relationships were consistently seen as the closest, most responsive, and most effective relationship type for fulfilling emotional needs, we found that people consider a strong PSR—someone they have never met (e.g., YouTube creator, a celebrity) and who may not even exist (e.g., fictional character)—as closer, and more effective at fulfilling their emotional needs than an acquaintance they interact with dyadically (i.e., their weak two-sided relationship).

Our findings were not limited to people thinking that PSRs might be helpful for emotion regulation in hypothetical situations. In Study 3, when their need for validation and responsiveness was acutely activated, people with high self-esteem defensively affirmed that their PSRs were responsive to their needs, in the same way that people typically do with strong two-sided relationships^30,32^. This study suggests that people acutely believe that their PSRs will provide them with positive and reliable support, despite the psychological irony that such one-sided relationships are not capable of being responsive, validating or (instrumentally) supportive. Notably, people with low self-esteem did not show the same compensatory effect. Past research finds that LSEs chronically doubt their close others^36^, but our study suggests that this chronic doubt does not extend to PSRs. LSEs in our study perceived greater responsiveness from their PSRs than HSEs when they were not threatened, and this did not change under threat. This suggests that the extent to which LSEs perceive their PSRs as responsive is potentially more stable and less contingent on acute changes in their emotional needs.

Alternatively, we may have been unable to detect a change in LSEs perceptions of the responsiveness of their PSR due to a ceiling effect. Our findings are consistent with past research whereby LSEs chronically rely more extensively on their online networks^37^. Indeed, exploratory analyses in Studies 1 and 2, which did not involve an acute threat, found that LSEs were more likely than HSEs to feel their PSRs are responsive to their needs. Future research should explore whether this chronic reliance on PSRs stems from the fact that LSEs derive more benefits than HSEs from engaging with PSRs to fulfil emotional needs not met by others.

The current findings complement existing research demonstrating the important social and psychological roles of PSRs (e.g.,^4,5,7^). Instead of simply acting as surrogates that people use to fill a gap in their social network^13^, our research finds that PSRs are in fact integral to social portfolios. It might not be possible for PSRs to reach out with personally tailored emotional support, but people nonetheless feel that they can rely on PSRs in times of need. This disconnect between what people feel and what is really possible matters less than one might expect. In romantic relationships for example, perceptions of a partner’s commitment and satisfaction are a more powerful predictor of how positively people feel about their relationships than the partner’s actual attitudes^38^. Indeed, humans appear to have not evolved sufficiently to differentiate between “real” and “imaginary” parasocial friendships^39^. Thus, *perceiving* a one-sided relationship as being able to meet one’s needs should be reality enough to feel validated and supported.

Future research could investigate the mechanisms through which PSRs effectively regulate emotions, and how these compare to the mechanisms at work in more traditional two-sided relationships, particularly given our findings that PSRs were seen as more effective at fulfilling emotional needs than weak in-person ties. There is an interesting paradox to consider as to which type of relationship (two-sided vs. PSR) offers someone the most reliability and control to get what they need emotionally and instrumentally. In some ways, two-sided relationships have clear advantages. For example, someone might realize in a two-sided interaction that rather than validating frustration like they usually would, in this instance they should attempt to cheer up their friend. A PSR is not capable of such dynamic social adaptation and agentic responsiveness because the interaction is driven through one-sided engagement. Someone could therefore get stuck in a downward emotional spiral without the dyadic intervention to pull them out. Likewise, interactions with many PSRs cannot change over time: a favorite movie character can only cheer you up with the same joke so many times before it loses its charm. However, two-sided relationships also have disadvantages; sometimes they are not willing or able to offer the type of support that people want^40,41^, sometimes they offer support that is ultimately unhelpful despite best intentions^42^, and ultimately it is impossible to control or perfectly predict how another person will respond from day to day. Taken together, this means real people will inevitably let us down at some point^17,18^. One-sided relationships may avoid some of these issues, because they can be selected based on their perceived ability to provide specific kinds of support. Further, PSRs can be more reliable than two-sided relationships: existing in people’s imaginations^13^ means they are just an imaginary phone-call away and cannot recoil from a surprising disclosure, or actively reject you in a time of need. These differences may have important consequences for well-being and emotion regulation over time, as well as for understanding how PSRs contribute to social portfolios more broadly speaking.

As media changes, the types of PSRs people can form change with it. These changes may happen faster than human cognition is able to adapt to (e.g.,^39,43^). Indeed, we did not find any differences in outcomes for people who had a PSR with a YouTube content creator compared to those who had a PSR with a more traditional type of social surrogate (e.g., a celebrity; see Study 2). Thus, although people might have more opportunities to interact with some PSRs (e.g., YouTubers) than others (e.g., fictional characters), we did not find any evidence to suggest that people are experiencing these PSRs in categorically different ways. Nonetheless, another important line of future research is to understand the extent to which the type of PSR influences the processes observed in this paper.

The continuous evolution of social media also means that people can have real and simulated interaction with PSRs (e.g., a YouTube creator responding to a person’s comment; a Twitter account for a fictional character retweeting a person’s joke), and interact with a broader community of fans who share the same PSR. Fandoms are strongly associated with PSRs, but differ to them in meaningful ways (e.g.,^44^). Fandoms are associated with well-being and identity formation (^45,46^), independent of the PSR that connects them. Thus, future research could focus on understanding whether fandoms influence the perceived ability of PSRs to fulfil emotional needs effectively, and whether some of this need fulfilment is provided by the fandoms in the absence of the PSR.

The current studies are limited in that they focus on how much people feel PSRs can fulfill emotional needs relative to close and weak two-sided ties, without putting these sources of emotional regulatory support in direct competition. Future research could examine what happens when people are socially excluded, and then are given the opportunity to interact with either strong or weak two-sided relationships, a PSR, or nothing, and then measure how well people felt their emotional needs had been met in that interaction. This would help provide a more nuanced understanding of whether people simply *believe* their PSRs would be capable of addressing these important emotional needs, or whether they are actually effective. Therefore, further research is needed in order to better clarify when PSRs can act as one-to-one replacements for “real” two-sided strong and weak relationships, and to clarify how interacting with PSRs versus strong/weak two-sided relationships can help people spend their social currency more efficiently.

Finally, it is important to note that several of the effects observed across our studies were relatively small, particularly the moderating effects of self-esteem in Study 3. Caution should therefore be used when interpreting the generalizability of these findings across different contexts, and further research and replications are encouraged to understand the reliability and boundary conditions of our findings. However, as recommended by Götz and colleagues (^47^), small effects still contribute to the cumulative understanding of complex psychological phenomena.

## Conclusion

People form PSRs with real and fictional people. These PSRs may never be able to physically reach out and offer people care and support, but that does not prevent them from cheering people up when they are sad, or amplifying people’s moments of happiness. Far from being a last resort for the socially disconnected, PSRs have an important role within people’s social portfolios, and are a valuable social resource that can help fulfil emotional needs.

### Supplementary Information

Supplementary Information.

## Data Availability

The datasets analyzed during the current study are available in the project’s Open Science Framework repository, https://osf.io/epmvf.
