# Author Correction: People perceive parasocial relationships to be effective at fulfilling emotional needs

**DOI:** 10.1038/s41598-024-60558-w

**Published:** 2024-05-01

**Authors:** Shaaba Lotun, Veronica M. Lamarche, Ana Matran‑Fernandez, Gillian M. Sandstrom

**Affiliations:** 1 Departmentof Psychology, University of Essex, Colchester, Essex CO4 3SQ UK; 2 Brain-Computer Interfaces and Neural Engineering Lab, School of Computer Science and Electronic Engineering, University of Essex, Colchester, CO4 3SQ UK; 3 Schoolof Psychology, University of Sussex, Brighton, Sussex BN1 9RH UK

Correction to: *Scientifc Reports* 10.1038/s41598-024-58069-9, published online 08 April 2024

The original version of this Article omitted an affiliation for Shaaba Lotun. The correct affiliations are listed below.

Department of Psychology, University of Essex, Colchester, CO4 3SQ, UK.

Brain-Computer Interfaces and Neural Engineering Lab, School of Computer Science and Electronic Engineering, University of Essex, Colchester, CO4 3SQ.

In addition, Ana Matran-Fernandez was incorrectly affiliated with ‘Department of Psychology, University of Essex, Colchester, Essex CO4 3SQ, UK’. The correct affiliation is listed below.

Brain-Computer Interfaces and Neural Engineering Lab, School of Computer Science and Electronic Engineering, University of Essex, Colchester, CO4 3SQ.

The original Article has been corrected.

