# Supplementary material for: People perceive parasocial relationships to be effective at fulfilling emotional needs
Source: Sci Rep. 2024 Apr 8;14:8185. doi: 10.1038/s41598-024-58069-9 (PMC11002006; doi:10.1038/s41598-024-58069-9)
Supplement: Supplementary file 1 — Supplementary Information. [file 41598_2024_58069_MOESM1_ESM.docx]

**People perceive parasocial relationships to be effective at fulfilling emotional needs**

The pilot study (Study 0) was not pre-registered. Pre-registrations (Studies 1-3), materials, data, and analysis syntax are available on OSF: <https://osf.io/epmvf>.

**Pilot Study (Study 0)**

We examined the extent to which people think PSRs are effective for emotion regulation, and how their effectiveness compares to that of two-sided strong and weak relationships that typically make up social networks.

Method

**Participants**

We recruited 147 participants in person at a social media convention (VidCon, London*)* in February 2019 (104 identified as female, 41 as male, and 2 in another way, aged 16-75; *M*_age_ = 25 years, *SD* = 10). We recruited as many people as we could in 6 hours. A sensitivity analysis (1–β = 0.80, α = .05) using G*Power (Faul et al., 2007) suggests that our sample can detect effects of *f* ≥ .13.

**Procedure**

Participants were asked to nominate one target for each of three types of relationships: a strong two-sided relationship (“someone you are very close to, someone who you know really well (and knows you really well), and **someone who you confide in** or talk to about yourself, or your problems”), a weak two-sided relationship (“someone you are not very close to, who you don't know very well, but someone who you consider a friend, but would be unlikely to confide in”), and a strong PSR (“**a YouTube creator** that you watch and feel like you 'know' the most”). YouTube creators were chosen as parasocial targets because VidCon is a creator convention, and thus provides access to participants who frequently engage with these types of PSRs. Participants reported on how effectively each target would fulfil their emotional needs, how close they felt to the target, and how responsive the target is.

Measures

**Emotional need fulfilment***.* Participants rated the extent to which the three social targets helped them regulate 10 emotions (α’s = .88-.96, for the various targets). Items were measured on a 10-point scale (1 = *least effective*; 10 = *most effective*). We assessed the seven items from Cheung et al. (2015) (e.g., cheering up when sad, amplifying anger). We also report results that include three exploratory items, tailored to the context of PSRs (reducing loneliness, validating identity, providing entertainment).

**Relationship responsiveness and closeness***.* We included exploratory measures of perceived responsiveness and closeness. Four items assessed the perceived responsiveness of each social target, (e.g., ‘how understood/liked/respected do you feel by [X]’*;* α’s = .82-.91), and three items assessed how close participants felt with each social target (*‘*how close do you feel with [X]’; α’s = .73-.86). Both were assessed on a 10-point scale (1 = *not very*, 10 = *very*).

**Well-being.** We included an exploratory measure of subjective well-being. Participants completed a 4-item happiness measure ((Lyubomirsky & Lepper, 1999), a 5-item satisfaction with life measure (Diener et al., 1985), and a 3-item loneliness measure (Hughes et al., 2004). For each measure, we computed an average, z-transformed the averages, and finally averaged the z-scores to create a well-being index (α = .72).

**Self-esteem***.* We included self-esteem as an exploratory covariate to help account for differences in how reliable people feel their social networks are in meeting their needs (e.g., Murray et al., 2008). Participants completed a 10-item measure of self-esteem (α = .87; Rosenberg, 1965), assessed on a 7-point scale (1 = *strongly disagree*, 7 = *strongly agree*).

Additional measures were assessed (e.g., affiliation motivations, frequency of contact, interaction anxiety, mental well-being, personality); see OSF for full materials.

**Results**

**Emotional need fulfillment**

To test for differences between social targets, we ran a repeated measures ANOVA for each dependent measure. There was a significant effect of target on emotional need fulfilment, *F*(2, 292) = 162.37, *p* < .001, $\eta_{p}^{2}$ *=* .53, with a Least Significant Difference (LSD) post-hoc test confirming that all three targets significantly differed from each other, *p’s* < .001 (see Figure S1). As expected, participants perceived their strong two-sided relationships as the most effective at fulfilling emotional needs. They felt that their strong PSRs would help them fill their emotional needs better than their weak two-sided relationships.

The same pattern of results emerged when we used the 10-item measure of emotional need fulfillment, with our three exploratory items.

**Figure S1**

*Differences between targets on perceived need fulfilment, responsiveness, and closeness for the Pilot Study*

*Note.* Error bars reflect the standard error.

**Relationship responsiveness and closeness**

Repeated measures ANOVAs revealed that participants perceived the social targets to differ in terms of perceived responsiveness, *F*(2, 292) = 242.38, *p* < .001, $\eta_{p}^{2}$ *=* .62, and perceived closeness, *F*(2, 292) = 288.82, *p* < .001, $\eta_{p}^{2}$ *=* .66 (see Figure 1). LSD post-hoc testing found the same pattern as with emotional need fulfillment: two-sided strong relationships were perceived as the most responsive and close, followed by strong PSRs, and then two-sided weak relationships, *p’s* < .001.

**Well-being**

We used linear regression to examine how the perceived ability of each target to fulfil emotional needs uniquely contributes to well-being when controlling for self-esteem. Self-esteem is associated with greater reported well-being and an overconfidence that others are willing to meet one’s needs (Leary et al., 1995). We therefore wanted to rule out the alternative explanation that people high in self-esteem are simply more likely to confidently overperceive that people would help them fulfil their needs, regardless of the type of relationship. Perceived effectiveness of strong two-sided relationships was positively associated with well-being, *b* = .06, CI_95_ = [.004, .11], *β* = .13, *t*(141) = 2.11, *p =* .04. However, neither perceived effectiveness of strong PSRs, *b* = .04, CI_95_ = [-.002, .08], *β* = .11, *t*(141) = 1.88, *p =* .06, nor of weak two-sided relationships, *b* = .02, CI_95_ = [-.02, .06], *β* = .05, *t*(141) = .87, *p* = .39, was significantly associated with well-being above and beyond the perceived effectiveness of strong two-sided relationships.

**Study 1**

**Method**

Measures

**Well-being.** We used the same happiness, satisfaction with life and loneliness measures as in the pilot study (but this time the loneliness measure was assessed on 7-point scale, 1 = *never*, 7 = *always*). Average scores on each measure were z-transformed and then averaged to create a well-being index (α = .76).

**Self-esteem***.* We used the same measure of self-esteem as in the pilot study (α = .91).

**Results**

**Emotional need fulfillment**

Results for each individual emotional need are reported in Table S1.

**Well-being**

We used linear regression to examine how the perceived ability of each target to fulfill emotional needs uniquely contributes to well-being when controlling for self-esteem. Perceived effectiveness of strong two-sided relationships was significantly associated with well-being, *b* = .08, CI_95_ = [.05, .10], *β* = .10, *t*(1647) = 5.67, *p <* .001 (consistent with the pilot study), as was perceived effectiveness of weak two-sided relationships, *b* = .03, CI_95_ = [.01, .05], *β* = .04, *t*(1647) = 2.53, *p* = .01 (inconsistent with the pilot study). Further, perceived effectiveness of strong PSRs was significantly associated with well-being, but in a negative direction, *b* = -.03, CI_95_ = [-.05, -.01], *β* = -.05, *t*(1647) = -2.74, *p =* .01 (inconsistent with the pilot study, where the association was positive but not statistically significant). Perceived effectiveness of weak PSRs (not measured in the pilot study) was not significantly associated with well-being, *b* = -.01, CI_95_ = [-.03, .01], *β =* -.01, *t*(1647) = -.68, *p* = .49.

**Table S1**

*Differences between targets on individual need fulfilment items for Study 1*

| Item | Strong Two-sided  M (SD) | Strong PSR  M (SD) | Weak Two-sided  M (SD) | Weak PSR  M (SD) | F |
| --- | --- | --- | --- | --- | --- |
| I'm anxious and want to calm down e.g. before an interview | 5.86 (1.43) | 5.07 (1.64) | 2.83 (1.66) | 2.30 (1.61) | F(3, 4929) = 2,132.75, p < .001, $\eta_{p}^{2}$ *=*.57 |
| I'm sad and want cheering up e.g. after a break up | 6.13 (1.28) | 5.25 (1.66) | 3.10 (1.72) | 2.46 (1.73) | F(3, 4935) = 2,135.43, p < .001, $\eta_{p}^{2}$ *=*.57 |
| I'm angry and want calming down e.g. after being in a fight | 5.72 (1.50) | 4.67 (1.67) | 2.64 (1.58) | 2.19 (1.54) | F(3, 4938) = 2,014.30, p < .001, $\eta_{p}^{2}$ *=*.55 |
| I'm angry and want this anger amplified e.g. before a competition | 4.44 (2.00) | 3.22 (1.82) | 2.98 (1.85) | 2.55 (1.95) | F(3, 4938) = 334.95, p < .001, $\eta_{p}^{2}$ *=*.17 |
| I've received good news and want to savour this happiness e.g. after getting a promotion | 6.34 (1.13) | 5.04 (1.76) | 3.74 (1.80) | 2.49 (1.74) | F(3, 4935) = 1,918.64, p < .001, $\eta_{p}^{2}$ *=*.54 |
| I'm feeling guilty and want to reduce this feeling e.g. after hurting someone's feelings | 5.71 (1.42) | 4.59 (1.71) | 2.65 (1.55) | 2.07 (1.42) | F(3, 4938) = 2,209.64, p < .001, $\eta_{p}^{2}$ *=*.57 |
| I'm embarrassed and want to reduce this e.g. after tripping over | 5.45 (1.61) | 4.66 (1.77) | 2.78 (1.66) | 2.18 (1.53) | F(3, 4941) = 1,637.70, p < .001, $\eta_{p}^{2}$ *=*.50 |

**Study 2**

**Method**

Measures

**Well-being.** We used the same happiness (α = .87), satisfaction with life (α = .90) and loneliness (α = .90) measures as in Study 1. Average scores on each measure were z-transformed and then averaged to create a well-being index (α = .82).

**Self-esteem***.* We used the same measure of self-esteem as in the pilot study and Study 1 (α = .93).

**Results**

**Emotional need fulfillment**

Results for each individual emotional need are reported in Table S2.

**Table S2**

*Differences between targets on individual need fulfilment items for Study 2*

| Item | Strong Two-sided  M (SD) | Strong PSR  M (SD) | Weak Two-sided  M (SD) | Weak PSR  M (SD) | F |
| --- | --- | --- | --- | --- | --- |
| I'm anxious and want to calm down e.g. before an interview | **5.98 (1.31)**  *5.92 (1.33)* | **4.69 (1.65)**  *5.18 (1.46)* | **2.95 (1.63)**  *3.23 (1.59)* | **2.49 (1.57)**  *2.12 (1.47)* | **F(3, 1035) = 442.17, p < .001,** $\boldsymbol{\eta}_{\boldsymbol{p}}^{\boldsymbol{2}}$ ***=*.56**  *F(3, 462) = 230.86, p < .001,* $\eta_{p}^{2}$ *=.60* |
| I'm sad and want cheering up e.g. after a break up | **6.11 (1.26)**  *6.35 (1.13)* | **4.87 (1.59)**  *5.41 (1.40)* | **3.13 (1.75)**  *3.50 (1.60)* | **2.64 (1.69)**  *2.33 (1.64)* | **F(3, 1035) = 429.91, p < .001,** $\boldsymbol{\eta}_{\boldsymbol{p}}^{\boldsymbol{2}}$ ***=*.56**  *F(3, 462) = 271.30, p < .001,* $\eta_{p}^{2}$ *=.64* |
| I'm angry and want calming down e.g. after being in a fight | **5.82 (1.35)**  *5.86 (1.39)* | **4.38 (1.69)**  *4.74 (1.58)* | **2.83 (1.60)**  *2.82 (1.54)* | **2.36 (1.54)**  *2.08 (1.34)* | **F(3, 1035) = 409.64, p < .001,** $\boldsymbol{\eta}_{\boldsymbol{p}}^{\boldsymbol{2}}$ ***=*.54**  *F(3, 462) = 244.11, p < .001,* $\eta_{p}^{2}$ *=.61* |
| I'm angry and want this anger amplified e.g. before a competition | **4.70 (2.00)**  *4.45 (2.01)* | **3.64 (1.92)**  *3.76 (1.83)* | **2.57 (1.54)**  *2.71 (1.62)* | **2.43 (1.70)**  *2.90 (2.07)* | **F(3, 1035) = 140.83, p < .001,** $\boldsymbol{\eta}_{\boldsymbol{p}}^{\boldsymbol{2}}$ ***=*.29**  *F(3, 459) = 29.39, p < .001,* $\eta_{p}^{2}$ *=.16* |
| I've received good news and want to savour this happiness e.g. after getting a promotion | **6.36 (1.08)**  *6.48 (.86)* | **4.75 (1.70)**  *5.43 (1.30)* | **3.49 (1.70)**  *4.06 (1.64)* | **2.71 (1.65)**  *2.45 (1.69)* | **F(3, 1035) = 449.03, p < .001,** $\boldsymbol{\eta}_{\boldsymbol{p}}^{\boldsymbol{2}}$ ***=*.57**  *F(3, 462) = 279.50, p < .001,* $\eta_{p}^{2}$ *=.65* |
| I'm feeling guilty and want to reduce this feeling e.g. after hurting someone's feelings | **5.82 (1.38)**  *5.86 (1.32)* | **4.23 (1.70)**  *5.01 (1.53)* | **2.71 (1.52)**  *2.95 (1.59)* | **2.31 (1.47)**  *2.05 (1.40)* | **F(3, 1035) = 469.83, p < .001,** $\boldsymbol{\eta}_{\boldsymbol{p}}^{\boldsymbol{2}}$ ***=*.58**  *F(3, 462) = 259.71, p < .001,* $\eta_{p}^{2}$ *=.63* |
| I'm embarrassed and want to reduce this e.g. after tripping over | **5.71 (1.48)**  *5.67 (1.52)* | **4.23 (1.74)**  *5.03 (1.50)* | **2.88 (1.68)**  *3.24 (1.62)* | **2.44 (1.60)**  *2.08 (1.37)* | **F(3, 1035) = 338.73, p < .001,** $\boldsymbol{\eta}_{\boldsymbol{p}}^{\boldsymbol{2}}$ ***=*.50**  *F(3, 462) = 227.45, p < .001,* $\eta_{p}^{2}$ *=.60* |

*Note*. Statistics for YouTube PSR’s are in bold, and statistics for non-YouTube PSR’s are in italics.

**Well-being**

We used linear regression to examine how the perceived ability of each target to fulfill emotional needs uniquely contributes to well-being when controlling for self-esteem. As in the pilot study and Study 1, perceived effectiveness of strong two-sided relationships was positively associated with well-being, *b* = .11, CI_95_ = [.06, .16], *β* = .13, *t*(495) = 4.61, *p <* .001. Meanwhile, perceived effectiveness of all other targets did not predict well-being above and beyond strong two-sided relationships: weak two-sided relationships, *b* = -.01, CI_95_ = [-.04, .03], *β* = -.01, *t*(495) = -0.32, *p* = .75; strong PSRs, *b* = -.001, CI_95_ = [-.04, .04], *β* = -.002, *t*(495) = -0.06, *p =* .95; weak PSRs, *b* = -.02, CI_95_ = [-.06, .02], *β =* -.03, *t*(495) = -0.89, *p* = .37.

**Meta-analysis of the pilot study and Studies 1-2**

In order to estimate the size of the key effects, we conducted internal meta-analyses. We ran paired sample t-tests to compare each pair of social targets; comparisons involving weak PSRs were only available in Studies 1 and 2, whereas all other comparisons use data from the pilot study as well. We meta-analyzed effect size *d*’s (Borenstein et al., 2009), which were computed using the average of the standard deviations for each pair of targets, as suggested by Cumming (2013; p.291). We conducted the meta-analyses using a random-effects model in Cumming’s meta-analysis module in the Exploratory Software for Confidence Intervals. Complete meta-analytic effect sizes are reported in Table S3.

Strong two-sided relationships were rated as the most effective at fulfilling emotional needs and were perceived as more responsive and closer than all other targets, meta-analytic *d*’s ≥ 0.82 (see Table S1). Strong PSRs were perceived as significantly more effective than weak two-sided relationships at fulfilling emotional needs, meta-analytic *d* = 1.24, CI_95_ = [1.13, 1.35], and were perceived as significantly closer, meta-analytic *d* = 0.75, CI_95_ = [0.38, 1.13], but did not differ significantly from them in perceived responsiveness, meta-analytic *d* = 0.52, CI_95_ = [-0.20, 1.23]. Weak PSRs were rated as less effective at emotion regulation, and perceived as less responsive and less close than all other targets, meta-analytic *d*’s ≥ 0.48.

**Table S3**

*Meta-analytic effect sizes, comparing each pair of targets*

|  |  | Need fulfilment | Responsiveness | Closeness |
| --- | --- | --- | --- | --- |
|  |  | mean diff /  meta-analytic d | mean diff /  meta-analytic d | mean diff /  meta-analytic d |
| strong vs. weak | study 1 | 2.8632 | 3.2571 | 3.5808 |
|  | study 2 | 2.7038 | 3.1527 | 3.5903 |
|  | study 3 | 2.7645 | 3.1123 | 3.8503 |
|  | meta-analysis | d = 2.26,  CI95 = [2.09, 2.44] | d = 2.63,  CI95 = [2.45, 2.80] | d = 3.29,  CI95 = [2.88, 3.70] |
|  |  |  |  |  |
| strong vs. strong-psr | study 1 | 0.8279 | 1.1796 | 1.8649 |
|  | study 2 | 1.0283 | 3.1067 | 3.0021 |
|  | study 3 | 1.2218 | 2.9526 | 2.9195 |
|  | meta-analysis | d = 0.82,  CI95 = [0.58, 1.07] | d = 1.85,  CI95 = [0.95, 2.76] | d = 2.28,  CI95 = [1.52, 3.04] |
|  |  |  |  |  |
| strong vs. weak-psr | study 2 | 3.342 | 4.7024 | 4.8759 |
|  | study 3 | 3.3684 | 4.7028 | 4.8963 |
|  | meta-analysis | d = 2.85,  CI95 = [2.63, 3.07] | d = 5.22,  CI95 = [4.85, 5.60] | d = 6.08,  CI95 = [5.65, 6.51] |
|  |  |  |  |  |
| weak vs. strong-psr | study 1 | 2.0353 | 2.0775 | 1.7159 |
|  | study 2 | 1.6755 | 0.046 | 0.5882 |
|  | study 3 | 1.5427 | 0.1597 | 0.9308 |
|  | meta-analysis | d = 1.24,  CI95 = [1.13, 1.35] | d = 0.52,  CI95 = [-0.20, 1.23] | d = 0.75,  CI95 = [0.38, 1.13] |
|  |  |  |  |  |
| weak vs. weak-psr | study 2 | 0.6382 | 1.5497 | 1.2856 |
|  | study 3 | 0.6039 | 1.5905 | 1.046 |
|  | meta-analysis | d = 0.48,  CI95 = [0.38, 0.58] | d = 1.3,  CI95 = [1.17, 1.43] | d = 1.06,  CI95 = [0.85, 1.26] |
|  |  |  |  |  |
| strong-psr vs. weak-psr | study 2 | 2.3137 | 1.5957 | 1.8738 |
|  | study 3 | 2.1466 | 1.7502 | 1.9768 |
|  | meta-analysis | d = 1.68,  CI95 = [1.53, 1.83] | d = 1.23,  CI95 = [1.10, 1.36] | d = 1.68,  CI95 = [1.53, 1.84] |

**References**

Borenstein, M., Hedges, L. V., Higgins, J., & Rothstein, H. R. (2009). *Introduction to meta-analysis*. Wiley Online Library. http://onlinelibrary.wiley.com/doi/10.1002/9780470743386.refs/summary

Cumming, G. (2013). *Understanding the new statistics: Effect sizes, confidence intervals, and meta-analysis*. Routledge.

Diener, E., Emmons, R. A., Larsen, R. J., & Griffin, S. (1985). The satisfaction with life scale. *Journal of Personality Assessment, 49*(1), 71-75. https://doi.org/https://doi.org/10.1207/s15327752jpa4901_13

Hughes, M. E., Waite, L. J., Hawkley, L. C., & Cacioppo, J. T. (2004). A short scale for measuring loneliness in large surveys: Results from two population-based studies. *Research on Aging, 26*(6), 655-672. https://doi.org/https://doi.org/10.1177/0164027504268574

Lyubomirsky, S., & Lepper, H. S. (1999). A measure of subjective happiness: Preliminary reliability and construct validation. *Social Indicators Research, 46*(2), 137-155. https://doi.org/https://doi.org/10.1023/A:1006824100041
